# Supplementary material for: Out-of-pocket expenditures and financial risks associated with treatment of vaccine-preventable diseases in Ethiopia: A cross-sectional costing analysis
Source: PLoS Med. 2023 Mar 10;20(3):e1004198. doi: 10.1371/journal.pmed.1004198 (PMC10004560; doi:10.1371/journal.pmed.1004198)
Supplement: S1 STROBE checklist — (DOCX) [file pmed.1004198.s001.docx]

**Webappendix 1: STROBE Checklist**

STROBE Statement—Checklist of items that should be included in reports of ***cross-sectional studies***

|  | Item No | Recommendation | Section and line numbers |
| --- | --- | --- | --- |
| **Title and abstract** | 1 | (*a*) Indicate the study’s design with a commonly used term in the title or the abstract | Abstract, under Methods and findings, 1^st^ paragraph. |
|  |  | (*b*) Provide in the abstract an informative and balanced summary of what was done and what was found | Abstract, under Methods and findings, 1^st^ paragraph. |
| Introduction | | | |
| Background/rationale | 2 | Explain the scientific background and rationale for the investigation being reported | Introduction, paragraphs 1-6. |
| Objectives | 3 | State specific objectives, including any prespecified hypotheses | Introduction, 7^th^ paragraph. |
| Methods | | | |
| Study design | 4 | Present key elements of study design early in the paper | Methods, 1^st^ paragraph. |
| Setting | 5 | Describe the setting, locations, and relevant dates, including periods of recruitment, exposure, follow-up, and data collection | Methods, study area and population, 1^st^ paragraph.  Methods, study sites and participant recruitment, 1^st^ paragraph.  Methods, data collection, paragraphs 1-2. |
| Participants | 6 | (*a*) Give the eligibility criteria, and the sources and methods of selection of participants | Methods, study sites and participant recruitment, paragraphs 4-5. |
| Variables | 7 | Clearly define all outcomes, exposures, predictors, potential confounders, and effect modifiers. Give diagnostic criteria, if applicable | Methods, data analysis, 1^st^ and 3^rd^ paragraphs. |
| Data sources/ measurement | 8* | For each variable of interest, give sources of data and details of methods of assessment (measurement). Describe comparability of assessment methods if there is more than one group | Methods, data analysis, 1^st^ and 3^rd^ paragraphs. |
| Bias | 9 | Describe any efforts to address potential sources of bias | Methods, data analysis, 3^rd^ paragraph. |
| Study size | 10 | Explain how the study size was arrived at | Methods, study sites and participant recruitment, 3^rd^ paragraph. |
| Quantitative variables | 11 | Explain how quantitative variables were handled in the analyses. If applicable, describe which groupings were chosen and why | Methods, data analysis, paragraphs 1-5 |
| Statistical methods | 12 | (*a*) Describe all statistical methods, including those used to control for confounding | Methods, data analysis, last paragraph. |
|  |  | (*b*) Describe any methods used to examine subgroups and interactions | Methods, data analysis, last paragraph. |
|  |  | (*c*) Explain how missing data were addressed | NA |
|  |  | (*d*) If applicable, describe analytical methods taking account of sampling strategy | NA |
|  |  | (*e*) Describe any sensitivity analyses | Methods, data analysis, 3^rd^ paragraph. |
| Results | | | |
| Participants | 13* | (a) Report numbers of individuals at each stage of study—eg numbers potentially eligible, examined for eligibility, confirmed eligible, included in the study, completing follow-up, and analysed | Results, sociodemographic characteristics, 1^st^ paragraph. |
|  |  | (b) Give reasons for non-participation at each stage | NA |
|  |  | (c) Consider use of a flow diagram | NA |
| Descriptive data | 14* | (a) Give characteristics of study participants (eg demographic, clinical, social) and information on exposures and potential confounders | Results, sociodemographic characteristics, 1^st^ paragraph and Table 1 |
|  |  | (b) Indicate number of participants with missing data for each variable of interest | NA |
| Outcome data | 15* | Report numbers of outcome events or summary measures | Results, magnitude of OOP expenditures, 1^st^ paragraph and Figure 1; Results, Cases of CHE and IHE, 1^st^ paragraph, Table 4 and figure 2. |
| Main results | 16 | (*a*) Give unadjusted estimates and, if applicable, confounder-adjusted estimates and their precision (eg, 95% confidence interval). Make clear which confounders were adjusted for and why they were included | Results, magnitude of OOP expenditures, 1^st^ and 2^nd^ paragraphs and Table 2; Results, Cases of CHE and IHE, 2^nd^ paragraph, Table 5 |
|  |  | (*b*) Report category boundaries when continuous variables were categorized | NA |
|  |  | (*c*) If relevant, consider translating estimates of relative risk into absolute risk for a meaningful time period | NA |
| Other analyses | 17 | Report other analyses done—eg analyses of subgroups and interactions, and sensitivity analyses | Results, Cases of CHE and IHE, 1^st^ paragraph, Table 4 |
| Discussion | | | |
| Key results | 18 | Summarise key results with reference to study objectives | Discussions, 1^st^ and 2^nd^ paragraphs. |
| Limitations | 19 | Discuss limitations of the study, taking into account sources of potential bias or imprecision. Discuss both direction and magnitude of any potential bias | Discussions, 6^th^ paragraph. |
| Interpretation | 20 | Give a cautious overall interpretation of results considering objectives, limitations, multiplicity of analyses, results from similar studies, and other relevant evidence | Discussions, 7^th^ paragraph |
| Generalisability | 21 | Discuss the generalisability (external validity) of the study results | Discussions, 5^th^ and 6^th^ paragraphs. |
| Other information | | | |
| Funding | 22 | Give the source of funding and the role of the funders for the present study and, if applicable, for the original study on which the present article is based | Funding from Gavi, the Vaccine Alliance. The funder had no role in the design, conduct and analysis of the study or approval and decision to submit the manuscript for publication. The views expressed are those of the authors and not necessarily those of the funder. |

*Give information separately for exposed and unexposed groups.

**Note:** An Explanation and Elaboration article discusses each checklist item and gives methodological background and published examples of transparent reporting. The STROBE checklist is best used in conjunction with this article (freely available on the Web sites of PLoS Medicine at http://www.plosmedicine.org/, Annals of Internal Medicine at http://www.annals.org/, and Epidemiology at http://www.epidem.com/). Information on the STROBE Initiative is available at www.strobe-statement.org.
