# Supplementary material for: Out-of-pocket expenditures and financial risks associated with treatment of vaccine-preventable diseases in Ethiopia: A cross-sectional costing analysis
Source: PLoS Med. 2023 Mar 10;20(3):e1004198. doi: 10.1371/journal.pmed.1004198 (PMC10004560; doi:10.1371/journal.pmed.1004198)
Supplement: S1 PROSPECTIVE protocol — (PDF) [file pmed.1004198.s002.pdf]

Webappendix 2: Prospective protocol (abridged)

1. OBJECTIVE OF THE RESEARCH PROJECT

1.1. General Objective

The study aims to assess the economic impact of accessing health services for selected vaccine preventable diseases in Ethiopia

1.2. Specific objectives

1.2.1. To estimate the direct costs (medical and non-medical) related to out-of-pocket (OOP) health expenditure for an episode of selected VPDs in Ethiopia.

1.2.2. To estimate indirect economic costs (productivity loss) to households associated with seeking health care for an episode of selected VPDs in Ethiopia.

1.2.3. To assess direct and indirect cost distributions by socio-economic status.

2. MATERIALS AND METHODS

We will conduct a cross-sectional study to assess the household-level economic consequences of seeking healthcare for an episode of a potential VPD in children and young adolescents below age 15. The study will employ a health-facility-based face-to-face exit interview coupled with a phone call interview within 2-to-4 weeks of the initial interview to capture potential relevant consequences that might occur after the first interview until the child recovers from the illness episode. We will use a structured questionnaire (appendix 1) adapted from previous similar studies.

We expect to complete the data collection within a period of 10 weeks over November 2020-January 2021. Each participant would have potentially 2 to 3 encounters with the research team over a period of 2 to 4 weeks to allow time for the child full recovery from the illness episode. The face-to-face interviews would take about 30 minutes while follow-up phone calls would be expected to last <= 10 minutes.

We plan to recruit a total of 1,773 subjects that meet the inclusion criteria from a sample of public health facilities using recruitment script (appendix 2). The only data to be potentially

extracted from patient medical records is diagnosis to avoid misclassification of cases by parental caregivers.

Inclusion criteria: caregivers of adolescents under 15 years of age with a confirmed diagnosis of bacterial meningitis or caregivers of children under 5 years of age clinically diagnosed with either pneumonia, diarrhea, measles, or whooping cough (pertussis) having just received healthcare for these conditions via either outpatient or inpatient care services.

Exclusion criteria: caregivers of children who are potentially eligible but who also have other comorbidities. For example, a child with measles and conjunctivitis will be included while a child with pneumonia and congenital heart diseases will be excluded. A list outlining major complications from the primary diagnoses will be provided to data collectors to facilitate consistent recruitment process. Moreover, caregivers of seriously ill patients who die from the illness episode will be excluded.

Participants' recruitment

Participants will be recruited from a systematically selected set of 54 public health facilities (table 1): 18 hospitals, 26 health centers, and 10 health posts. These will be located in the capital city of Addis Ababa and in five (out of) nine Ethiopian regions: Oromia, Amhara, Southern Nations Nationalities and Peoples (SNNP), Somali, and Afar. In each facility, responsible healthcare providers (also hired and trained as data collectors for this study) will proactively identify eligible subjects from pediatric outpatient departments and inpatient wards based on patient diagnosis documented on respective medical charts. Outpatient cases will be enrolled consecutively when an IMCI-trained clinician identifies them as having diarrhea, pneumonia or measles until the sample size quota is obtained. Similarly, severe cases of pneumonia, severe diarrhea, severe measles or meningitis cases will be consecutively enrolled from paediatric inpatient units after the physician in charge confirms

the diagnosis of severe pneumonia, severe diarrhea, severe measles or meningitis. We expect to recruit 10-20% of the study sample from inpatient wards.

Reliable epidemiological data showing the magnitude and distribution of VPDs is missing for Ethiopia. To effectively design the study, we relied on several expert-informed assumptions. For example, the incidence of VPDs in a given locality is mainly determined by the size of the local susceptible population, vaccine coverage, and case fatality. Taking such considerations into account, we will maximize our operational efficiency without compromising the representativeness of the study population. We also considered feasibility (local logistical constraints) of data collection, time efficiency, and limited financial resources available. As a result, we included 20 hospitals, 25 health centers, and 10 health posts selected from five regions and Addis Ababa. We included public facilities as, according to the 2016, 75% of those seeking care were taken to public facilities (predominantly to health centers) and private healthcare providers represented only 15% of the services provided.

Ethiopia is administratively organized into nine regions and two city administrations. Regions display wide variations in immunization coverage levels. Therefore, first, we selected regions that performed below national average on DPT3, PCV13, or measles vaccine coverage, or on coverage of fully immunized children. This led to Oromia, SNNP, Amhara, Afar, Somali, Harari, and Gambella regions to be selected. Since Addis Ababa serves as a referral destination for the whole Ethiopia, we also included 3 hospitals from Addis Ababa as well, which leaves total of 17 hospitals, 25 health centers, and 10 health posts to be allocated across the regions.

To do this regional allocation, we used the relative population size of under-15 children in these six regions. Based on the 2018 population projections, these regions account for 91% of the total Ethiopian population and 93% of the under-15 children and young adolescents nationwide. Accordingly, Oromia, Amhara, SNNP, Somali, and Afar housed 44%, 22%, 24%, 7%, 2% of under-15 year-olds and thus, would contribute proportionally for the selection of each facility type. For example, 7, 11, and 4 of the hospitals, health centers, and heath posts, respectively, were selected from the Oromia region.

To come up with a sampling frame that would more likely take us to high burden areas within regions, we used two datasets. Firstly, the coverage of DPT3 disaggregated by zone (administrative units between regions and districts). This data is drawn from the Global Burden of Disease Study and shows the percentage of children in each zone that have received DPT3 in 2016. This allows identifying possible "hot spots" within regions. Secondly, the master health facility list in Ethiopia from the Service Provision Assessment survey conducted in 2014. The master facility list provides the list of health facilities by type in each region with respective zonal codes that we could link to data from (a). Hence, we could derive five sampling frames of hospitals located within close proximity of the lowest DPT3 coverage zone for each region. Subsequently, we randomly selected the required number of hospitals in each region, and once this was complete, we prepared five sampling frames of list of health centers for each region by listing all the health centers located in the same town as the hospitals included in the final sample and followed the same random selection process to select the final list of health centers to be included. For the health posts, regional coordinators will identify those posts closest to the already randomly selected health centers to maximize operational efficiency.

Sample size calculation

For pneumonia, diarrhea (including potentially measles and pertussis patients), we estimated the difference in mean total cost across any two wealth quintiles to be US$5 and standard deviation of US$10. For meningitis, given its severity and possibly long hospital stays, the corresponding mean difference was assumed to be US$15 and standard deviation of US$32. Accordingly, with 80% power to detect such difference with 95% confidence, we would need 66 subjects per quintile for meningitis and 63 per quintile for each of the four conditions above. Assuming a 10% non-response rate, this would mean that we would need to recruit 385 cases of meningitis and 347 cases of pneumonia, diarrhea, measles, and pertussis. This would give a total sample size of 1670 subjects.

Data analysis

The expenditures in local currency units will be converted to USD using the prevailing exchange rate and proper equivalence scale adjustments (age, sex, and geography) will be done before analyzing household consumption expenditures. Descriptive statistical methods will be used to summarize the OOP direct medial expenditures, direct non-medical costs, and indirect costs.
