# Supplementary material for: Out-of-pocket expenditures and financial risks associated with treatment of vaccine-preventable diseases in Ethiopia: A cross-sectional costing analysis
Source: PLoS Med. 2023 Mar 10;20(3):e1004198. doi: 10.1371/journal.pmed.1004198 (PMC10004560; doi:10.1371/journal.pmed.1004198)
Supplement: S1 Data Tool — (PDF) [file pmed.1004198.s003.pdf]

ECONOMIC IMPACT OF VACCINE PREVENTABLE DISEASES ON HOUSEHOLDS IN ETHIOPIA

Webappendix 3: Data collection tool

To be filled upon completion of outpatient visit or discharge from inpatient admission

1_1 Region ______________ 1_2 District/Town __________________

1_3 Facility type: ____________

1) Hospital
2) Health center
3) Health post

1_4 Health facility name ______________ 1_5 Child's card number___________

1_6 Date of interview (dd/mm/yy)____/___/____

1_7 Interviewer Name ____________ 1_8 Time interview started _________

**Section A: Background and basic medical information**<br>In this section, I will ask you about some general information about yourself, the child, and the household the child, lives in.					
A_1_1	What is the diagnosis of the child <Name>?<br>***(copy from medical chart)***<br>***(for the interviewer)***		________________________________		
A_1_2	What type of medical service did the child receive?<br>***(for the interviewer)***<br>1) Outpatient<br>2) Inpatient*<br>****Inpatient care is defined as at least an overnight stay in the health facility***		________________________________		
A_1_3	If answer to Q 'A_1_2' is 'inpatient', what was the length of stay in the health facility?		_________________days (nights)		
A_1_4	How many people respond to this interview?		_______________		
A_1_5	What is the respondent's relationship with the child?<br>1) Father<br>2) Mother<br>3) Sibling<br>4) Other relatives<br>5) Neighbour<br>6) Other(describe)		1.Respondent_1<br><br>____________	2.Respondent_2<br><br>__________	3.Respondent_3<br><br>__________
A_1_6	How old is the respondent(s)?		____________	__________	__________

<table>
<tr><td>A_1_7</td><td>Sex of the respondent(s)
1) Male
2) Female</td><td></td><td colspan="2">_____________</td><td colspan="2">___________</td><td>___________</td></tr>
<tr><td>A_1_8</td><td>What is the educational status of respondent(s)?
1) No education
2) Read and write
3) Primary education or less
4) Secondary education or less
5) Above secondary education</td><td></td><td colspan="2">_____________</td><td colspan="2">___________</td><td>___________</td></tr>
<tr><td>A_1_9</td><td>What is the child’s date of birth?</td><td></td><td colspan="5">(dd/mm/yy)___/___/____
99) don’t know_________</td></tr>
<tr><td>A_1_10</td><td>If the date of birth is not known, what is the age of the child?</td><td></td><td colspan="5">____________in months
____________in years</td></tr>
<tr><td>A_1_11</td><td>What is the sex of the child?
1) Male
2) Female</td><td></td><td colspan="5">__________________</td></tr>
<tr><td>A_1_12</td><td>What’s the child’s education status?
(if child is school age and enrolled in any school)</td><td></td><td colspan="5">_______________________
99) don’t know _________
999) not reported _______</td></tr>
<tr><td>A_1_13</td><td>Where is the child’s residential address?</td><td></td><td colspan="5">1.Region ___________________________
2.District/Town ______________________
3.Kebele ___________________________
99) Don’t know
999) Not reported _________________</td></tr>
<tr><td>A_1_14</td><td>How is the residential address categorized?
1) Urban
2) Rural
99) Don’t know</td><td></td><td colspan="5">___________________</td></tr>
<tr><td>A_1_15</td><td>How many people live in the household where the child lives including the child?

*household is defined as person(s) who occupy a dwelling (i.e., share the same shelter, cooking, washing, and sleeping facilities); this may or may not be a family.</td><td></td><td colspan="5">Total number of people_______________</td></tr>
<tr><td rowspan="3">A_1_16</td><td rowspan="3">Please specify the age, sex, and the relationship of each household member to the child?</td><td rowspan="3"></td><td></td><td>a.age</td><td>b.sex</td><td colspan="2">c. relationship</td></tr>
<tr><td>1.</td><td></td><td></td><td colspan="2"></td></tr>
<tr><td>2.</td><td></td><td></td><td colspan="2"></td></tr>
</table>

	1. father<br>2. mother<br>3. sibling<br>4. grand parent<br>5. other (describe)		3.			
			4.			
			5.			
			6.			
			7.			
			8.			
			9.			
			10.			
**Section B: Medical care prior to the current health facility visit/admission.**<br>In this section, I will ask you about treatment services the child received, if any, prior to the current health facility visit/admission.						
B_1_1	For the current episode of illness, has your child received medical treatment from any source before the current health facility outpatient visit/admission?		__________<br>1) Yes<br>2) No<br>99) Don't know			
	If 'Yes' to Q 'B_1_1', please answer questions 'B_1_2 to B_1_5'<br><br>***If 'No' to Q 'B_1_1', please skip to Section C***					
B_1_2	If yes to Q 'B_1_1', how many outpatient visits/admissions did the child had before the current health facility visit/admission?	a.outpatient visit _____________<br>b.inpatient admission__________				
B_1_3	Please specify type of the healthcare providers visited in each of the previous visits?<br><br>***(Consider visit_1 as the most recent)***<br><br>1) Government hospital<br>2) Health center<br>3) Health post<br>4) Private hospital<br>5) Private clinic<br>6) NGO hospital or clinic<br>7) Drug store or pharmacy<br>8) Traditional healer		b. Visit_1	c. Visit_2	d.Visit_3	

	9) Others (describe)				
B_1_4	What type of service did the child received during each of the previous health facility visits?<br><br>1) Outpatient<br>2) Inpatient*<br>99) Don't know<br><br>***inpatient is defined as at least an overnight stay in a health facility***		____________	___________	___________
B_1_5	Please specify the date(s) for each visit/admission.<br>**(dd/mm/yy)**		___/___/___	___/___/___	___/___/___
**Section C: Out-of-pocket payments for the health services received and the sources of financing households used to cope with the payments.**<br>In this section, I am going to ask you whether and how much you paid for the services you received both at the current health facility and the visits prior to the current, if you had any, as well as what sources you tapped into to mobilize these funds.					
C_1_1	For each of the previous and the current healthcare provider visits, did you spend any amount out-of-pocket for receiving medical services for the child's current episode illness?<br><br>(*This includes all expenses including direct medical (e.g. drugs) and direct non-medical costs (e.g. transportation)*<br><br>***(Consider visit 1 as the more recent health facility visit prior to the current visit)***	a.current visit<br><br>_____________<br>_<br>1) Yes<br>2) No<br>99) Don't know	b.visit_1<br><br>_____________<br>1) Yes<br>2) No<br>99) Don't know	c.visit_2<br><br>___________<br>1) Yes<br>2) No<br>99) Don't know	d.visit_3<br><br>___________<br>1) Yes<br>2) No<br>99) Don't know
C_1_2	If the answer to Q 'C_1_1' is 'NO', what was the	____________	___________	__________	___________

	reason you did not pay?<br>1) Expenses covered by CBHI<br>2) Expenses covered by other insurance scheme<br>3) Services were exempted<br>4) Household is beneficiary of the fee-waiver scheme<br>5) Health facility was near and therefore, we did not have any transportation or other related expenses<br>6) Other (describe)				
	If the answer to Q 'C_1_1' is 'YES', please report how much you spend on each of the following items for each of the visits.<br>***(Consider visit 1 as the more recent health facility visit prior to the current visit)***<br>*All expenses to be reported in Ethiopian birr*	a.current visit	b.visit_1	c.visit_2	d.visit_3
C_1_3	Total expenses				
C_1_4	Consultation/registration fee				
C_1_5	Laboratory tests/investigation				
C_1_6	Drugs				
C_1_7	Hospital bed day **(only for those who had been admitted)**				
C_1_8	Transportation **(to and from the health facility)**<br>***For the 'current visit' consider what mode of transport you will be using to return back home.**				

C_1_9	Extra food cost expenses<br>*(food bought for the child and/or for caregivers who accompanied the child to the health facility)*				
C_1_10	Additional expenses for care giver<br>*(such as compensation, if any)*				
C_1_11	Others expenses (amount)				
C_1_12	Others expenses (describe)				
C_1_13	Overall, how much money have you spent to get healthcare for receiving medical services from the start of this episode of illness till today?<br><br>(*Some households may not remember all the details above)		____		
C_2	**What source(s) of finance did you use to cover those expenses (B_1_5 to B_1_14)?**<br>***(specify amount from each source as appropriate)***	a.current visit	b.visit_1	c.visit_2	d.visit_3
C_2_1	Current income<br>**(income earned within the last 30days)**				
C_2_2	Own saving				
C_2_3	Asset sale				
C_2_4	Borrowed				
C_2_5	Support or gifts from family/friends				
C_2_6	Reimbursement by insurance				
C_2_7	Others source (specify)				

Section D: Time and wage loss related to seeking health services.

In this section, I am going to ask you about how much time and wage each of the care givers lost while seeking care for the child.

D_1_1	How many care-givers did the child had who were responsible to care for the child on a regular basis during this episode of illness? ***regular means**	a.current visit<br>__________	b.visit_1<br>__________	c.visit_2<br>__________	d.visit_3<br>_________
D_2	Specify the relationship of each care giver to the child and how much time each spent while taking care of the child or seeking care for illness?	a. current visit	b. visit_1	c.visit_2	d.visit_3
D_2_1_1	Caregiver<br>Relation with the child	______________	______________	_____________	______________
D_2_1_2	Total time while caring for the child at home and on the road to and in the health facility	____hour(s)<br>____day(s)	____hour(s)<br>____day(s)	____hour(s)<br>____day(s)	____hour(s)<br>____day(s)
D_2_1_3	Time spent at home caring for the child	____hour(s)<br>____day(s)	____hour(s)<br>____day(s)	____hour(s)<br>____day(s)	____hour(s)<br>____day(s)
D_2_1_4	Time spent in the health facility while waiting to receive care	____hour(s)	____hour(s)	____hour(s)	____hour(s)
D_2_1_5	Time spent in the health facility with the health care provider while receiving care	____hour(s)<br>____day(s)	____hour(s)<br>____day(s)	____hour(s)<br>____day(s)	____hour(s)<br>____day(s)
D_2_1_6	Time spent traveling to and from health facility	____hour(s)<br>____day(s)	____hour(s)<br>____day(s)	____hour(s)<br>____day(s)	____hour(s)<br>____day(s)
D_2_2_1	Caregiver 2<br>Relation with the child	______________	______________	_____________	____________
D_2_2_2	Total time while caring for the child at home and on the road to and in the health facility	____hour(s)<br>____day(s)	____hour(s)<br>____day(s)	____hour(s)<br>____day(s)	____hour(s)<br>____day(s)
D_2_2_3	Time spent at home caring for the child	____hour(s)<br>____day(s)	____hour(s)<br>____day(s)	____hour(s)<br>____day(s)	____hour(s)<br>____day(s)
D_2_2_4	Time spent in the health facility while waiting to receive care	____hour(s)	____hour(s)	____hour(s)	____hour(s)

D_2_2_5	Time spent in the health facility while receiving care	____hour(s)<br>____day(s)	____hour(s)<br>____day(s)	____hour(s)<br>____day(s)	____hour(s)<br>____day(s)
D_2_2_6	Time spent traveling to and from health facility	____hour(s)<br>____day(s)	____hour(s)<br>____day(s)	____hour(s)<br>____day(s)	____hour(s)<br>____day(s)
D_2_3_1	Caregiver 3<br><br>Relation with the child	______________	______________	______________	______________
D_2_3_2	Total time while caring for the child at home and on the road to and in the health facility	____hour(s)<br>____day(s)	____hour(s)<br>____day(s)	____hour(s)<br>____day(s)	____hour(s)<br>____day(s)
D_2_3_3	Time spent at home caring for the child	____hour(s)<br>____day(s)	____hour(s)<br>____day(s)	____hour(s)<br>____day(s)	____hour(s)<br>____day(s)
D_2_3_4	Time spent in the health facility while waiting to receive care	____hour(s)	____hour(s)	____hour(s)	____hour(s)
D_2_3_5	Time spent in the health facility while receiving care	____hour(s)<br>____day(s)	____hour(s)<br>____day(s)	____hour(s)<br>____day(s)	____hour(s)<br>____day(s)
D_2_3_6	Time spent traveling to and from health facility	____hour(s)<br>____day(s)	____hour(s)<br>____day(s)	____hour(s)<br>____day(s)	____hour(s)<br>____day(s)
D_3_1	Please specify what each care-giver would have done with the time they spent taking care of the sick child (see B_5) if they did not have to take care of the sick child?<br>1. Work(paid)<br>2. Work (home and non-paid)<br>3. Attend school<br>4. Leisure<br>5. Spend with family and friends<br>6. Other (specify)	a.current visit	b.visit_1	c.visit_2	d.visit_3
	a. Caregiver_1	_____________	_____________	_____________	_____________
	b. Caregiver_2	_____________	_____________	_____________	_____________
	c.Caregiver_3	_____________	_____________	_____________	_____________

D_3_2	For caregivers with paid jobs, did you lose wages while taking care of the sick child or seeking care for the illness during this particular episode?<br>1) Yes<br>2) No	a.current visit	b.visit_1	c.visit_2	d.visit_3
	a. Care-giver_1	____________	____________	____________	____________
	b. Care-giver_2	____________	____________	____________	____________
	c. Care-giver_3	____________	____________	____________	____________
D_3_4	If 'yes' to Q 'B_6_1', how much wage did you lose during each of the visits?	a.current visit	b.visit_1	c.visit_2	d.visit_3
	a. Care-giver_1	___________	__________	__________	_________
	b. Care-giver_2	___________	__________	_________	_________
	c.Caregiver_3	___________	__________	__________	_________

**Section E: Household consumption expenditures.**<br>In this section, I will ask you about household expenses for essential consumptions including food. Please report all expenses in Ethiopian birr.			
	On average, how much does your household spend on the following items in a given month:		
E_1_1	Food and supplies<br>(e.g. raw ingredients, any semi-cooked/cooked/food/snack/sweets etc.) **per month**		______________________
E_1_2	Does the household consume any home-produced food or goods during the (e.g. wheat, rice, fruits, vegetables, milk, milk products etc.)		☐ Yes<br>☐ No
E_1_3	If yes, specify what you produced?		______________________<br>______________________
E_1_4	If you were to buy the same food items (C_1_3) from the market, how much would		

	you had to pay on average for an equivalent quantity of what you produced and consumed?		________________
E_1_5	Does the household received and consumed any food item and supplies as a gift or as an in-kind compensation for any work delivered?		☐ Yes<br>☐ No
E_1_6	If yes, please specify what you received?		___________________________
E_1_7	If you were to buy the same food items and supplies (C_1_6) from the market, how much would you have to pay on average for an equivalent quantity of what you received and consumed?		________________
E_1_8	Utilities<br>*(electricity, water, telephone) per month*		________________
E_1_9	Education<br>*(School for children or self) (per month/per term/per year)*<br>*(circle the appropriate time frame and specify the respective amount in the box provided)*		________________
E_1_10	Rent<br>(house/land/shop) per month		________________
E_1_11	Tobacco and alcohol		
E_1_12	Health care<br>*(for the household) last months*		________________
E_1_13	Health insurance		________________
E_1_14	Goods and utensils<br>*(for the household use) per year*		________________
E_1_15	Clothes<br>*(for the household) per year*		________________
E_1_16	Maintenance of bicycle, scooters, Bajaj, cars *(maintenance and fuel) per month*		________________
E_1_17	Replacements of household appliances<br>*(stove, fridge, food processor, etc.) Per month*		________________
E_1_18	Reimbursement of loan<br>*(for the household use) per month*		________________
E_1_19	Travel(transportation) costs<br>*(for the household) per month*		________________
E_1_20	Others expenses (specify type)		_________________
E_1_21	Others expenses (amount)		__________________

**Section F: Household income**<br>In this section, I will ask you about the income of the head of the household and all the other income generating and contributing members of the household from official employment, rent or sale of productive assets, and remitence or gifts received on regular basis. Please report all expenses in Ethiopian birr.			
F_1_1	Total monthly average income of the head of the household (primary income generator) from employment?		
F_1_2	Total monthly average household income of all the other economically active and contributing members of the household from employment?		
F_1_3	Total monthly average income of the household from sale or rent of economically productive assets owned by the household?		___________________
F_1_4	Total monthly average household income from gifts or remittance to members of the household?		___________________
**Section G: Household assets**<br>In this section, I will ask you about the house you live in and household's possessions.			
G_1_1	Does the household own a residential house?<br>1.Yes<br>2.No		___________
G_1_2	If 'yes' to Q 'G_1_1', does the household live in this house?<br>1.Yes<br>2.No		___________
G_1_3	If 'no' to Q 'G_1_1', does the household live in a rental house?<br>1.Yes<br>2.No		___________
G_1_4	If 'yes' to Q 'G_1_3', how much house rent does the household you pay per month?		
G_1_5	How many rooms does the house where the household lives have?		________________
G_1_6	How many of these rooms(G_1_5) are used for sleeping?		________________
G_1_7	What is the main source of drinking water for the household?<br>1. Pipe within the household compound<br>2. Public tap<br>3. Well within the household compound<br>4. Public well<br>5. Other(describe)		________________

G_1_8	What source of energy does the household use for cooking?<br>1.Electricity<br>2.Gas<br>3.Kerosine<br>4.Wood<br>5.Coal<br>6.Other (describe)		_______________
G_1_9	What kind of toilet facility does the household use?<br>1.Private flush<br>2.Public flush<br>3.Private pit toilet<br>4.Public pit toilet<br>5.Other (describe)		________________
G_1_10	Does the household has electricity?		1.Yes<br>2.No
G_1_11	Does the household or any member of the household own these items?<br><br>1.Yes<br>2.No		1. Chair________<br>2. Table________<br>3. Bed_________<br>4. Radio________<br>5. Television____<br>6. Refrigerator____
G_1_12	How many of the following animals does the household own?		1.Cattle___________<br>2.Horse, donkey, or mule ________<br>3.Goats_______<br>4.Sheep_______<br>5.Chicken______<br>6.Camels_______<br>7.Other(describe)_______
G_1_13	How many of these goods does the household possess?		1.Bicycle_________<br>2.Motor cycle_____<br>3.Bajaj____________<br>4. Car or truck_____________<br>5. Tractor ____________<br>6. Plowing machine ________<br>7. Other(sepecify)___________
G_1_14	Do members of the household own plot of land for farming?		1.Yes<br>2.No
G_1_15	If 'yes' to Q 'G_1_13', how large is the land?		________in hectare

Thank you for successfully completing the interview. If you are willing, we would like to contact you within 2-4weeks time to capture any additional out-of-pocket payments you make or time lost related to the child's illness on subsequent days until the child fully recovers. If you are willing, please provide us with your phone number that we can use to reach out to you or the other care-givers.

Name and phone number of respondent_1 ____

Name and phone number of respondent_2 ____

Name and phone number of respondent_3 ____

Time the interview ended______________________

Signature of the interviewer____________________

THE SECTION BELOW IS TO BE USED FOR THE FOLLOW UP PHONE INTERVIEWS

Section H: Expenses during the follow up period.
Call to be made 2 weeks after the first face-to-face interview.

H_1_1	Has the child completed all the medications prescribed and fully recovered from the illness?<br>1.Yes<br>2.No, still recovering<br>3.No, child passed away		________
	If the answer to Q 'H_1_1' is 'No, still recovering', call back again in 2-week time.		
	If the answer to Q 'H_1_1' is 'No, child passed away', thank them for their participation so far and conclude the call.		
	If the answer to Q 'H_1_1' is 'yes', proceed to the following questions.		

The following questions pertain to what happened between the first (face-to-face) interview and now.

H_1_2	Did the child receive any additional medical service after the first interview?	1.Yes<br>2.No
H_1_3	How many encounters/visits did the child had?	____________

H_1_4	What type of service did the child received during each of the previous health facility visits?<br><br>1.Outpatient<br>2.Refill of medication<br>3.Inpatient*<br><br>***inpatient is defined as at least an overnight stay in a health facility**	a.visit_1	b.visit_2	c.visit_3
H_1_5	For each of these encounters/visits, did you spend any amount out-of-pocket for receiving medical services?<br><br>(*This includes all expenses including direct medical (e.g. drugs) and direct non-medical costs (e.g. transportation)*<br><br>***(Consider visit 1 as the most recent visit**)*	a.visit_1<br><br>_______________	b.visit_2<br><br>______________	c.visit_3<br><br>_____________
	If the answer to Q 'H_1_5' is 'Yes', please report how much you spent on each of the following items for each of the visits.<br>***(Consider visit 1 as the more recent health facility visit prior to the current visit**)*<br>*All expenses to be reported in Ethiopian birr*	a.visit_1	b.visit_2	c.visit_3
H_1_6	Total expenses			
H_1_7	Consultation/registration fee			
H_1_8	Laboratory tests/investigation			
H_1_9	Drugs			
H_1_10	Hospital bed day **(only for those who had been admitted)**			
H_1_11	Transportation **(to and from the health facility)**<br>***For the 'current visit' consider what mode of transport you will be using to return back home.**			

H_1_12	Extra food cost expenses<br>*(food bought for the child and/or for caregivers who accompanied the child to the health facility)*			
H_1_13	Additional expenses for care giver<br>*(such as compensation, if any)*			
H_1_14	Others expenses (amount)			
H_1_15	Others expenses (describe)			
H_1_16	Overall, how much money have you spent to get healthcare for receiving medical services from the from the first interview till today?<br><br>(*Some households may not remember all the details above)		____________________________________	

Thank you again for completing the follow up phone call interview successfully. Please provide any remark or feedback you may have related to the study.

____
____
____
____
____
____

Name of the interviewer ____________________

Signature __________________

Date _____________________
