# Supplementary material for: Out-of-pocket expenditures and financial risks associated with treatment of vaccine-preventable diseases in Ethiopia: A cross-sectional costing analysis
Source: PLoS Med. 2023 Mar 10;20(3):e1004198. doi: 10.1371/journal.pmed.1004198 (PMC10004560; doi:10.1371/journal.pmed.1004198)
Supplement: Attachment — Submitted filename: Review ResponsePM.docx [file pmed.1004198.s004.docx]

November 19, 2022

*PLOS Medicine* Editors

Dear Editors,

We are submitting our revised manuscript entitled **"Out-of-pocket expenditures and financial risks associated with treatment of vaccine-preventable diseases in Ethiopia" (PMEDICINE-D-22-02567R1)** to *PLOS Medicine.*

We would like to thank the editor and reviewers for the constructive comments and suggestions which have helped to strengthen our work substantially. Below, we have provided a detailed point-by-point response to all comments and suggestions raised in the review. We have also edited the manuscript to conform to *PLOS Medicine’s* requirments and formatting.

Thank you very much for your consideration of our manuscript.

Sincerely,

Solomon Tessema Memirie, on behalf of the authors

**Editors’ comments**

GENERAL
Please address all editor and reviewer comments detailed below, in full
Your study combines observational and economic/cost analyses. Please review the following relevant guidance and report your study according to relevant reporting guidelines.  I have made some suggestions below.
Please ensure that the study is reported according to the STROBE guideline, and include the completed STROBE checklist as Supporting Information. Please add the following statement, or similar, to the Methods: "This study is reported as per the Strengthening the Reporting of Observational Studies in Epidemiology (STROBE) guideline (S1 Checklist)." The STROBE guideline can be found here: <http://www.equator-network.org/reporting-guidelines/strobe/> When completing the checklist, please use section and paragraph numbers, rather than page numbers.

**Authors’ reponse: We have included the sentence: "This study is reported as per the Strengthening the Reporting of Observational Studies in Epidemiology (STROBE) guideline (S1 Text)." Under Methods. Please see page 8, Lines 196-198.
We have also included the completed STROBE checklist as a Supporting Information document (S1 Text).**

Please ensure that you consider the following guidance, relevant to your study design, when reporting your cost analysis, provided at:
<http://www.equator-network.org/post_type=eq_guidelines&eq_guidelines_study_design=economic-evaluations&eq_guidelines_clinical_specialty=0&eq_guidelines_report_section=0&s=> 
Please also provide the relevant completed checklist. In the checklist please include sufficient text excerpted from the manuscript to explain how you accomplished all applicable items.

**Authors’ reponse: we purposedly did not fill the CHEERS checklist because it is not applicable to our study. Our analysis is not a full economic evaluation, rather it only focuses on patient costs and it does not compare two interventions in terms of costs and benefits as in a typical cost-effectiveness analysis study (which our study is not). Therefore, we considered not appropriate to include the CHEERS checklist in the reporting of our study.**

ABSTRACT
Please structure your abstract using the PLOS Medicine headings (Background, Methods and Findings, Conclusions). Please combine the Methods and Findings sections into one section, “Methods and findings”.

**Authors’ reponse: we have structured the abstract in compliance with *PLOS Medicine* requirements. Please see “Abstract” section pages 2-3.**

Abstract Methods and Findings:
Please ensure that all numbers presented in the abstract are present and identical to numbers presented in the main manuscript text.

**Authors’ reponse: we confirm that the numbers presented in the abstract are presented in the main manuscript section and are identical.**

Please explicitly state when data was collected from households and over what time frame if there was more than one data collection point (please include mean, SD and range)

**Authors’ reponse: the abstract section includes the time frame of the data collection. We have also included mean values and ranges as appropriate. Please see “Abstract” section, pages 2-3.**


Please explicitly state the main outcome measures

**Authors’ reponse: we have now explicitly stated the main outcome measures. Please see the Abstract, page 2, lines 51-52.**

Please quantify the main results with 95% CIs and p-values

**Authors’ reponse: when quantifying the main results, we have included 95% CIs and p-values. Please see the Abstract, page 2, lines 55-62.**

Please include any important dependent variables that are adjusted for in the analyses

**Authors’ reponse: we have now included important dependent variables that are adjusted for in the analyses. Please see the Abstract, page 2, lines 61-63.**

Would it be helpful to include the number of participants in the 995 households? I’m assuming 1 child per household (so 995 children) but these are contagious VPDs and households usually have more than one child so that is quite a big assumption to make (this is only explicitly clear by the time the reader reaches the results section of the main manuscript)

**Authors’ reponse: we have now modified the sentence to clarify this point on the number of participants. Please see the Abstract, page 2, line 50.**

Line 46: “..OOP costs” perhaps replace with expenditure for consistency? Please check and amend throughout (including in the main manuscript)

**Authors’ reponse: we have changed “costs” to “expenditures” uniformly throughout the manuscript.**

Line 55: “…13·3% of households…” please include denominator, for example, “…13.3% of 995 households…” or something similar

**Authors’ reponse: we have included the denominator in the text. Please see the Abstract, page 2, lines 59-60.**

In the last sentence of the Abstract Methods and Findings section, please describe the main limitation(s) of the study's methodology.

**Authors’ reponse: we have included the main limitation in the Abstract. Please see the abstract, page 2, lines 63-64.**


Abstract Conclusion
Line 58: “Conclusions: The OOP costs induced by VPDs are large in Ethiopia, especially for the poor and for patients requiring inpatient care.” Suggest “Conclusions: The OOP costs induced by VPDs are substantial in Ethiopia and disproportionately impact those with low-incomes and those requiring inpatient care.” Or something similar

**Authors’ reponse: we have modified the sentence as suggested above. It reads as follows: “The OOP expenditures induced by VPDs are substantial in Ethiopia and disproportionately impact those with low income and those requiring inpatient care.” Please see “Abstract”, page 3, lines 66-67.**


AUTHOR SUMMARY
At this stage, we ask that you include a short, non-technical Author Summary of your research to make findings accessible to a wide audience that includes both scientists and non-scientists. The Author Summary should immediately follow the Abstract in your revised manuscript. This text is subject to editorial change and should be distinct from the scientific abstract. Please see our author guidelines for more information: <https://journals.plos.org/plosmedicine/s/revising-your-manuscript#loc-author-summary>

**Authors’ reponse: we have included an “Author Summary” following the abstract. Please see “Author Summary”, page 4.**

METHODS and RESULTS
Please report the number of patients, households, etc and dates of recruitment

**Authors’ reponse: we have modified the results section to address the above points as follows: “A total of 995 patients (1 child per household). Please see “Results”, “Sociodemographic characteristics” first paragraph, line 368. The dates of recruitment have been described as follows: “All data were collected during May 1 to July 31 2021.” Please see last paragraph under “Methods”, “Data Collection” Line 305.**

Please account for all methods used in your study - please discuss how the data pertaining to wage-loss were collected/calculated in the methods section of the manuscript (see also reviewer comments below)

**Authors’ reponse: we have included the following under “Methods”, “Data collection”: “Additionally, we collected data on the employment status of each caretaker and on any income or wage losses while taking care of the sick child.” Page 11, lines 285-287. And under “Methods”, “Data analysis”: “We computed productivity losses for income or wage-earning family members as the sum of income losses of household members who engaged in the care of the sick child.” Page 12, lines 314-316.**

Please define the length of follow up (eg, in mean, SD, and range).

**Authors’ reponse: We have included the length of follow-up and modified the paragraph as follows: “A total of 995 patients (1 child per household) with a mean age of 1.9 years (95% confidence interval (CI): 1.7 to 2.0 years) were included in the final analysis, of whom 411 (41%) were rural residents (Table 1). We conducted follow-up phone calls after a mean duration of 14.4 days (SD:0.8 days and range 11-21 days) following the face-to-face interviews. All the households except one (994 out of 995) responded to the follow-up phone calls. We included 995 households in the final analysis” Page 14, lines 368-373.**

Please present numerators and denominators for percentages, at least in the Tables (see also comments below)

**Authors’ reponse: we have included numerators and denominators for percentages in the tables and in the relevant section in the text. Please see Tables 1 & 4.**

Please define "lost to follow-up" as used in this study. Other reasons for exclusion should be defined.

**Authors’ reponse: we included the following sentences on inclusion or exclusion criteria of study participants: “Children less than 5 years of age presenting with either diarrhea, pneumonia, pertussis or measles and without other concomitant illness were included in the study. Similarly, we included children less than 15 years who have meningitis and without other conditions. Children with multiple conditions and beyond the specified ages were excluded from the study.” Please see page 10, lines 270-274.**

Please specify whether informed consent was written or oral .

**Authors’ reponse: this has been specified as follows: “All study participants gave a written informed consent.” Please see page 12, lines 305-306.**

Did your study have a prospective protocol or analysis plan? Please state this (either way) early in the Methods section.

a) If a prospective analysis plan (from your funding proposal, IRB or other ethics committee submission, study protocol, or other planning document written before analyzing the data) was used in designing the study, please include the relevant prospectively written document with your revised manuscript as a Supporting Information file to be published alongside your study, and cite it in the Methods section. A legend for this file should be included at the end of your manuscript.
b) If no such document exists, please make sure that the Methods section transparently describes when analyses were planned, and when/why any data-driven changes to analyses took place. 
c) In either case, changes in the analysis-- including those made in response to peer review comments-- should be identified as such in the Methods section of the paper, with rationale.

**Authors’ reponse: we have now stated about the prospective protocol as follows: “The data collection and analysis were based on a prospective protocol (S2 Text).” Please see page 8, lines 198-199.**

**We have also attached the prospective protocol as S2 Text.**

For all observational studies, in the manuscript text, please ensure that you have clearly indicated: 
(1) the specific hypotheses you intended to test, 
(2) the analytical methods by which you planned to test them,
(3) the analyses you actually performed, and 
(4) when reported analyses differ from those that were planned, transparent explanations for differences that affect the reliability of the study's results. If a reported analysis was performed based on an interesting but unanticipated pattern in the data, please be clear that the analysis was data-driven.

**Authors’ reponse: we have included the completed STROBE checklist as a Supporting Information document (S1 Text).**

Line 235: “We employed face-to-face exit interviews…” this statement implies that there was an entry interview – my understanding is that this was the initial interview – for clarity perhaps instead “We employed face-to-face interviews.

**Authors’ reponse: we have now deleted “exit” and the sentence reads as follows: “We employed face-to-face interviews with….”, Page 11, line 276.**

TABLES
Table 1: please present denominators for percentages reported here.  For the row labelled: Mean household size – consider “Mean number of people per household”

**Authors’ reponse: we have included the actual numbers besides the percentages in table 1 and changed “Mean household size” to “Mean number of people per household”. Please see Table 1.**

Table 2: It’s not clear on only reviewing the figure what the “mean” numbers here refer to, please amend the table for improved clarity and accessibility to the reader. Similarly which piece of data presented do these p-values refer to?  Please also include the statistical tests used to determine these. were any factors adjusted for in these analyses? If so please specify which in the figure caption and include the unadjusted analyses.  Where p-values are reported please also report 95% CIs.  In the figure caption I would suggest replacing these symbols with superscript letters a, b, c and so on

**Authors’ reponse: to clarify Table 2, we have changed “Mean” to “Mean (95% CI) OOP expenditure” and included 95% confidence intervals. We have also added what statistical tests were used including adjustments for the independent variables and what the p-values referred to. We have also changed the captions to letters. Please see to Table 2.**

Table 3; “Average Loss” please add USD symbol in parentheses to make it clear what these numbers refer to.  Please remove “(standard deviation in parentheses)” from the table title and place in the caption/legend below

**Authors’ reponse: we have included the USD symbol and additional descriptions to improve the clarity of the table to readers as follows: “^h^Average loss refers to the average values for the five regions and Addis Ababa city administration combined.” Please see Table 3.**

Table 4: Please present numerators and denominators for percentages, at least in the Tables [not necessarily each time they're mentioned].

**Authors’ reponse: we have included numerators and denominators in the table. Please see Table 4.**

Table 5: were any factors adjusted for in these analyses? If so please specify which in the figure caption and include the unadjusted analyses

**Authors’ reponse: we have now added the following sentence under the table to clarify the point “The richest quintile, urban residence, health center visits, outpatient visits, diarrheal illness were reference values against which comparisons were made while adjusting for all the independent variables.” Please refer to Table 5.**

FIGURES
Throughout, to make the figures more accessible to those with colour blindness please avoid the use of red and/or green

**Authors’ reponse: we have now avoided red and green colors from figures. Please see figures 1&2.**

Figure 1 title: “…out-of-pocket medical expenditures” see previous comments re: Costs Vs Expenditure – please amend accordingly

**Authors’ reponse: we have changed “costs” to expenditures uniformly throughout the manuscript.**

Figure 2: please define - HH, OOP and (Int. US$)

**Authors’ reponse: we have defined the abbreviations. Please see figure 2.**

*** Throughout the manuscript there is inconsistent use of the terms “Expenditure” and “cost(s)” in figure and table titles and legends/captions please check and revise using one of these terms only ***

**Authors’ reponse: we have now used the term expenditure uniformly throughout the manuscript.**

DISCUSSION
Please ensure that your discussion is structured as follows: a short, clear summary of the article's findings; what the study adds to existing research and where and why the results may differ from previous research; strengths and limitations of the study; implications and next steps for research, clinical practice, and/or public policy; one-paragraph conclusion.

**Authors’ reponse: the discussion section has been edited to conform with the journal’s requirements. We included a clear summary of the study findings in the discussion section. Please see “Discussions”, first paragraph. Page 20, lines 480-483.**

Please remove the funding statement and declaration of interests statement from the end of the manuscript and include only in the manuscript submission form

**Authors’ reponse: we have removed the funding statement and declaration of interest statement from the manuscript.**

REFERENCES
For in text reference call outs, citations should be in square brackets, and preceding punctuation. Note the absence of spaces within square bracket, for example: – “asymptomatically [2,4].”
In the reference list, please ensure that no more than the first 6 authors are listed before et al where more than 6 authors have contributed to a study including in the supplementary references
Journal name abbreviations should be those found in the National Center for Biotechnology Information (NCBI) databases.
Please see our website for other reference guidelines <https://journals.plos.org/plosmedicine/s/submission-guidelines#loc-references>

**Authors’ reponse: we have edited text references, author listing, Journal name abbreviations based on the reference guideline.**

SUPPORTING INFORMATION
Please include the S1 checklist in-line with STROBE reporting and any other checklists from reporting guidance relevant to your cost analyses

**Authors’ reponse: STROBE checklist, Prospective protocol and Data collection tool are provided as supporting information**.

**Reviewers’ comments**

***Reviewer #1****:*

The manuscript that aims to estimate the extend of out-of-pocket payment and the associated catastrophic health expenditures due to selected VPDs in Ethiopia is well written and of potential interested to the readers of the journal.  I recommend that paper with proposed revisions should be considered for publication.

**Authors’ reponse: We thank the reviewer for her/his positive comments on our manuscript and the additional comments are addressed below.**

Major comments 
-	In the introduction or discussion section the authors should elaborate on the policy relevance of these types of studies in general and who the target audience is e.g. the cost could be  part of an input for return of investment analyses of vaccines and immunization programmes targeting Ministries of Finance.

**Authors’ response: Thank you for raising this important point. We have added the following into the introduction section of the manuscript: “Better estimates of household out-of-pocket expenditures for the treatment of VPDs in low- and middle-income countries allow for more precision in estimating the expected financial risk protection and return on investment of expanding access to vaccination.” Please see page 7, lines 184-187.**

-	In the discussion section the authors rightly highlight the study is biased towards urban well off study population. Would it be feasible  to extrapolate the study results if more marginalized populations from rural area would have included in the study?  If a quantification is not feasible, an analysis describing the direction of main parameters influencing the OOP and CHE results would be useful for decision makers and researchers in the field.

**Authors’ response: We have now added the following in the discussion section: “The data also indicates lower OOP expenses for rural residents for both outpatient and inpatient services as compared to their urban counterparts. Despite lower OOP expenses, rural households were more likely to incur CHE, possibly a function of households’ inability to cope with medical payments. The under representation of rural households in our study might underestimate the extent of CHE.
Please see page 22, lines 543-548.**

Minor comments
-	p 3. I suggest add the following key-words: costs (or costing)

**Authors’ response: we have now added costing as a key word. Please see “Key words”, page 3, line 73.**

-	p10, line 171. I suggest to also add average life expectancy for male and female in Ethiopia

**Authors’ response: we have included average life expectancy for male and female in Ethiopia. Please see page 8, line 204.**

-	p15 table 1. I guess the title of first column should be: Total number of cases by "city administration"

**Authors’ response: on page 15, rather than table 1, it is table 3. Therefore, our response is based on Table 3. We included the following sentence to elaborate on the title: “Average loss refers to the average values for the five regions and Addis Ababa city administration combined.” Please see Table 3, page 17.**

-	p19 line 375-376: the 10% vs 40% CHE threshold of OOP should be introduced in the methods section including the rationale behind the "usually used" 10% and why 40%? Is there any rationale? Why not adding a lower bound of 5% to give the full range of the sensitivity of CHE depending on the threshold?

**Authors’ response: We have modified the section as follows: “A case of CHE was calculated by computing OOP expenditures incurred minus any reimbursement from third-party payers divided by annual household expenditures [33,34]. Specifically, a household would incur CHE when OOP expenditures exceeded a certain threshold (10%. as commonly used) of consumption expenditures [15]. Additionally, we computed CHE cases using household non-food expenditures (capacity to pay defined as effective income net of subsistence spending) as the denominator and a 40% catastrophic threshold [15,35]. The thresholds represent severe disruptions in customary living standards of families as a result of health care spending. We also conducted a sensitivity analysis using a lower (5%) threshold of annual consumption expenditure” Page 12-13, lines 326-333 and 336-337.**

**We have now included CHE values at a 5% threshold of annual consumption expenditure. Please see Table 4.**

***Reviewer #2***

This study conducted a costing analysis trying to estimate the out-of-pocket (OOP) expenditure and productivity losses due to five vaccine-preventable diseases (VPDs) and assess the catastrophic health expenditures (CHE) caused by these VPDs. Overall, I think this study is well designed and conducted. The topic of the study is of interest and the results are very helpful to promote vaccination coverage in the low-and-middle income countries. Below are my specific comments.

**Authors’ response: We thank the reviewer for her/his constructive comments on our manuscript and the additional comments are addressed below.**

1.	"The link to the public data repository will be available after acceptance of the manuscript." I think the data repository needs to be made available before acceptance of the paper. However, I am not sure whether that would breach the confidentiality of the personal data.

**Authors’ response: The data set has been published in public repository with the following link:** [**https://doi.org/10.6084/m9.figshare.20425434**](https://doi.org/10.6084/m9.figshare.20425434)


2.	Line 143-144: would you specify the SDGs that vaccine can help realize?

**Authors’ response: we have included the specific SDGs vaccine can help realize and the sentence is modified as follows: “In short, vaccines could play a major role in the progressive realization of universal health coverage (UHC) and the Sustainable Development Goals (SDGs), specifically regarding SDG-1 (“To end poverty in all its forms everywhere”) and SDG-3 (“To ensure healthy lives and promote well-being for all at all ages”) targets in Ethiopia [21].” Page 7, line 167-171.**

3.	Line 188: how the two hospitals were selected? Were they selected randomly?

**Authors’ response: We thank the reviewer for pointing this out. The hospitals included were randomly selected. The sentence now reads as follows: “Since Addis Ababa serves as a referral destination for the whole Ethiopia, we included two randomly selected hospitals there.” Please see Page 9, lines 220-222.**

4.	Line 181-209: Is there a valid reason why you purposefully selected areas with high burden of the VPDs in the country? Will that selection bias your results?

**Authors’ response: Thank you for raising this important point. We have now added the following to clarify why high burden areas are selected. “Second, to obtain a sample that would more likely cover high burden areas and therefore maximize recruitment of adequate numbers of VPD cases within regions, we used two datasets.” Please see page 9, lines 228-230.**

**Additionally, under discussion and limitations we have included the following: “Third, we prioritized facilities from likely high burden areas to maximize recruitment of patients with VPDs. Even though we do not anticipate health care costs in public health facilities to differ based on the burden of VPDs, socioeconomic characteristics of the households may vary that could bias the study findings.” Please see page 22, lines 548-551.**

5.	Line 208-209: I understand public facilities used by most people. However, the 25% care seeking that takes place in private section may cost much more. Therefore, excluding those may severely underestimate the cost of seeking care for the VPDs on the national level.

**Authors’ response: We have included the following under limitation section of the Discussion: “Fourth, we included only public facilities which may underestimate the extent of OOP expenditures due to the likely higher costs in private facilities.” Please see page 22, lines 552-553.**

6.	Line 217: How were the mean 15 USD and SD of 32 USD were estimated or chosen?

**Authors’ response: we have clarified on the point as follows with inclusion of a reference to substantiate our assumption: “For bacterial meningitis, given the possibly long hospital stays associated (a threefold increase in mean length of stay for bacterial meningitis as compared to other conditions), the mean difference was assumed to be USD15 (with SD of USD32) [31].” Please see page 10, lines 251-254.**

7.	Line 296: ETB 8401 per year?

**Authors’ response: we have modified the sentence as follows to clarify the point: “We used the World Bank definition for extreme poverty (for low-income countries) as those living on less than $1·90 a day (in 2020 PPP), which was converted into a poverty line (per adult equivalent per year) of ETB 8401 [24].” Page 13, lines 342-344.**

8.	Line 301-302: Is that linear or logistic regression that you used? It's a bit confusing to me.

**Authors’ response: We have modified the sentence as follows: “We then ran linear regression models to assess the level of variation in the magnitude of OOP expenditures by wealth quintile, area of residence, facility type, region: logistic regressions were conducted to identify the drivers of differences in the odds of CHE, with firstly implementing bivariate models, and secondly multivariate models.” Please see page 13, lines 349-353.**

***Reviewer #3***

Dear Authors:

Thank you very much for the opportunity to review your manuscript entitled Out-of-pocket expenditures and financial risks associated with treatment of vaccine preventable diseases in Ethiopia. Your research paper explored out-of-pocket expenditures and financial risks of vaccine-preventable diseases from house-hold perspectives.

The paper is well-written with some room for improvement. Please see below for my comments.

**Authors’ response: We thank the reviewer for her/his positive comments on our manuscript. We have addressed the additional comments below.**

Line 194: The sample was taken from the high burden areas. Does this selection method bias the findings? Line 468 discusses some potential biases but the discussion is very limited to understand how the sampling technics might have contributed to biased results (or not). I recommend additional discussion on this.

**Authors’ response: Thank you for raising this important point. We have now added the following to clarify why high burden areas are selected. “Second, to obtain a sample that would more likely cover high burden areas and therefore maximize recruitment of adequate numbers of VPD cases within regions, we used two datasets.” Please see page 9, lines 228-230.**

**Additionally, under discussion and limitations we have included the following: “Third, we prioritized facilities from likely high burden areas to maximize recruitment of patients with VPDs. Even though we do not anticipate health care costs in public health facilities to differ based on the burden of VPDs, socioeconomic characteristics of the households may vary that could bias the study findings. Please see page 22, lines 548-551.**

Line 411: The discussion section does not include your assessment of vaccine's potential contributions to alleviate financial burdens. How would vaccination programs improve CHEs in percentages? (i.e. how would Table 4 look like after an implementation?) Are vaccination programs improve equity to health? If so, how would Table 2 look like once a program is implemented? While concrete numbers might not be found and out of scope for this study, I recommend some discussion regarding to return on investment for public policy stakeholders.

**Authors’ response: we thank the reviewer for the comment. We have now included the potential contribution of vaccines in our discussion section as follows: “Expanding access to vaccination has the potential to protect families from OOP expenditures and substantially reduce CHE and IHE associated with VPDs where such benefits would primarily accrue among the poorest [20].” Please see page 21, lines 510-512.**

Line 272: Wage-loss is first discussed in data analytics section. Please discuss how the data pertaining to this analysis are collected in the data collection section earlier in the manuscript. I suggest updating objectives to include measures used to identify productivity losses.

**Authors’ response: thank you for pointing out this omission. We have now included the following in the methods, data collection and analysis sections:**

**“Additionally, we collected data on the employment status of each caretaker and on any income or wage losses while taking care of the sick child.” Please see page 11, lines 285-287.**

**“We computed productivity losses for income or wage-earning family members as the sum of income losses of household members who engaged in the care of the sick child.” Please see page 12, lines 314-316.**
