# Supplementary material for: Out-of-pocket expenditures and financial risks associated with treatment of vaccine-preventable diseases in Ethiopia: A cross-sectional costing analysis
Source: PLoS Med. 2023 Mar 10;20(3):e1004198. doi: 10.1371/journal.pmed.1004198 (PMC10004560; doi:10.1371/journal.pmed.1004198)
Supplement: Attachment — Submitted filename: Review Response.docx [file pmed.1004198.s005.docx]

February 09, 2023

*PLOS Medicine* Editors

Dear Editors,

We are submitting our revised manuscript entitled **"Out-of-pocket expenditures and financial risks associated with treatment of vaccine-preventable diseases in Ethiopia" (PMEDICINE-D-22-02567R2)** to *PLOS Medicine.*

We would like to thank the editor and reviewers for the constructive comments and suggestions which have helped to strengthen our work substantially. Below, we have provided a detailed point-by-point response to all comments and suggestions raised in the review. We have also edited the manuscript to conform to *PLOS Medicine’s* requirments and formatting.

Thank you very much for your consideration of our manuscript.

Sincerely,

Solomon Tessema Memirie, on behalf of the authors

**Request from Editors:**

GENERAL
Thank you for your detailed and considerate responses to previous editor and reviewer comments. Please see below for further minor revisions which we require that you address, in full.

**Authors’ reponse: thank you very much. The additional comments are addressed in full below.**

TITLE
Please revise your title according to PLOS medicine’s style. Your title must be nondeclarative and not a question. It should begin with main concept if possible.  The study design ("A randomized controlled trial," "A retrospective study," "A modelling study," etc.) should be placed in the subtitle (ie, after a colon). Suggest – “Out-of-pocket expenditures and financial risks associated with treatment of vaccine preventable diseases in Ethiopia: a cross-sectional costing analysis.” Or something similar.

**Authors’ reponse: we have modified the title as suggested above. It reads as follows: “Out-of-pocket expenditures and financial risks associated with treatment of vaccine preventable diseases in Ethiopia: a cross-sectional costing analysis.” Please see “Title”, page 1, lines 2-3.**

STATISTICAL REPORTING
Throughout, consider the use of commas to separate upper and lower confidence limits instead of hyphens (as these can be confused with the presentation of negative values).

**Authors’ reponse: when presenting 95% CIs, we have now used commas instead of hyphens throughout the manuscript. Please see the Abstract, Results and Discussion sections.**

METHODS and RESULTS
Lines 285 onwards – we agree with the reviewer (see below) that this aspect of your methodology requires additional detail, please revise accordingly.

**Authors’ reponse: Thank you for raising this point again. We have included additional description to explain the data collection and analysis related to productivity losses as follows:**

***“We also collected data on caregivers (for a maximum of three) time losses and how that time would have been spent by the caregiver if she/he was not taking care of the sick child. We derived parents’ time losses by adding the time spent seeking care prior to outpatient consultation and/or inpatient admission to the duration of outpatient and/or inpatient visit. Additionally, we collected data on the employment status of each caregiver and if there were any losses in income or wage while taking care of the sick child.”***

**Please see Page 11, lines 283-289, under “Methods”, “Data collection”**

**And:**

***“We computed productivity losses for income or wage-earning family members as the sum of reported income losses of household members who engaged in the care of the sick child, i.e., reported income losses by caregiver-1 plus caregiver-2 plus caregiver-3.“***

**Please see Page 12, lines 315-318, under “Methods”, “Data analysis**

TABLES
Thank you for revising your tables which are now much clearer.  

To help facilitate transparent data reporting, PLOS Medicine requests that where adjusted analyses are reported, the unadjusted analyses are reported for comparison.  Please include and if not please clearly state why not.

**Authors’ reponse: we have now reported the results of the unadjusted analysis. Please see Table 5.**

Table 1 – to improve accessibility to the reader please adjust the table so that confidence limits are reported on a single line

**Authors’ reponse: we have now presented the confidence limits in a single line. Please see Table 1.**

Table 2 – your footnote key begins with the letter “c” but logically should begin with the letter “a” please revise

**Authors’ reponse: we have now adjusted the footnote key to begin with the letter “a”. Please see Table 2.**

Table 3 – as above, your footnote key begins with the letter “h” and should begin with the letter “a”.  Please revise throughout all tables

**Authors’ reponse: we have now adjusted the footnote key to begin with the letter “a”. Please see Table 3.**

Table 5 – as above, please replace “j” with “a”

**Authors’ reponse: we have now adjusted the footnote key to begin with the letter “a”. Please see Table 5.**

SOCIAL MEDIA
To help us extend the reach of your research, please provide any Twitter handle(s) that would be appropriate to tag, including your own, your coauthors’, your institution, funder, or lab. Please detail any handles you wish to be included when we tweet this paper, in the manuscript submission form when you re-submit the manuscript.

**Authors’ reponse: we have now included Twitteer handles in the manuscript submission form.**

Comments from Reviewers:

Reviewer #2: I think the authors have answered and addressed my and other reviewers' questions adequately. I believe the paper is acceptable for publication. Great work and congratulations to the authors! 

**Authors’ reponse: we thank the reviewer for reviewing our manuscript and for her/his positive comments.**

Reviewer #3: Dear Authors:

Thank you very much for the opportunity to review your manuscript entitled Out-of-pocket expenditures 
and financial risks associated with treatment of vaccine preventable diseases in Ethiopia once again. Your research paper explored out-of-pocket expenditures and financial risks of vaccine-preventable diseases from household perspectives.
The paper is well-written, and the authors provided detailed responses to the previous comments from reviewers and the editor.

**Authors’ reponse: we thank the reviewer for reviewing our manuscript and for her/his positive comments.**

I found one incomplete revision in response to my previous comment. Please address my additional request below:

Lines 285- 287, Thank you very much for adding the sentence to explain that data on productivity losses were collected.  However, the added sentence does not explain how the data were collected. Please include the method. Did interviewers directly ask this question? Please explain the data collection method. This applies to comments from the editor on "methods and results". Just stating "data were collected" do not sufficiently explain how exactly data collection was conducted. While the study protocol can be referred to, a concise explanation in the manuscript itself would be a great improvement in transparency.

**Authors’ reponse: Thank you for raising this point again. We have included additional description to explain the data collection and analysis related to productivity losses as follows:**

***“We also collected data on caregivers (for a maximum of three) time losses and how that time would have been spent by the caregiver if she/he was not taking care of the sick child. We derived parents’ time losses by adding the time spent seeking care prior to outpatient consultation and/or inpatient admission to the duration of outpatient and/or inpatient visit. Additionally, we collected data on the employment status of each caregiver and if there were any losses in income or wage while taking care of the sick child.”***

**Please see Page 11, lines 283-289, under “Methods”, “Data collection”**

**And:**

***“We computed productivity losses for income or wage-earning family members as the sum of reported income losses of household members who engaged in the care of the sick child, i.e., reported income losses by caregiver-1 plus caregiver-2 plus caregiver-3.“ Please see Page 12, lines 315-318, under “Methods”, “Data analysis”***
